# Molecular Detection and Identification of Plant-Associated *Lactiplantibacillus plantarum*

**DOI:** 10.3390/ijms24054853

**Published:** 2023-03-02

**Authors:** Magdalena Skotniczny, Paweł Satora

**Affiliations:** Department of Fermentation Technology and Microbiology, Faculty of Food Technology, University of Agriculture in Krakow, Balicka 122, 30-149 Kraków, Poland

**Keywords:** 16S rRNA, housekeeping genes, genotyping, WGS, qPCR, shotgun

## Abstract

*Lactiplantibacillus plantarum* is a lactic acid bacterium often isolated from a wide variety of niches. Its ubiquity can be explained by a large, flexible genome that helps it adapt to different habitats. The consequence of this is great strain diversity, which may make their identification difficult. Accordingly, this review provides an overview of molecular techniques, both culture-dependent, and culture-independent, currently used to detect and identify *L. plantarum*. Some of the techniques described can also be applied to the analysis of other lactic acid bacteria.

## 1. Introduction

*Lactiplantibacillus plantarum* (formerly *Lactobacillus plantarum*) is one of the most common lactic acid bacteria (LAB) isolated all over the world from spontaneously fermented plant material (e.g., sauerkraut, Chinese sauerkraut, kimchi, cucumbers, olives, carrot juice, tomatoes, leek, radish, *Carica papaya* leaves, mustard tuber, cocoa beans, chia sourdough [1,2,3,4,5,6,7,8,9,10,11,12,13]). It can also be found in dairy food, sausages, fish digestive tract/feces of animals, and humans or soil [14,15,16,17,18,19,20].

*L. plantarum* owes the ability to colonize such diverse habitats to its extraordinary genome [21]. It is larger than the genomes of most other lactic acid bacteria, ranging in size from 2.9 to 3.7 Mbp [22]. It is also characterized by high plasticity, mainly due to the existence of lifestyle adaptation islands. These are highly variable sets of genes, to a great extent related to sugar metabolism, which can be modified in response to environmental conditions [23]. As a consequence, this species is characterized by a very high genetic and phenotypic diversity, which is constantly reported by scientists. Comparative analysis of *L. plantarum* genomes isolated from different niches showed that isolates of plant origin, unlike those of dairy or animal origin, do not possess many environmental-specific genes [24]. For the above reasons, they are classified as generalists/nomads.

The aforementioned flexibility of *L. plantarum* explains its great ecological, technological, and thus also scientific and economic importance [25]. Years of research and industrial use as starter cultures and probiotics have resulted in the species getting the QPS status in the European Union and many strains have been notified as GRAS in the United States. Nevertheless, new isolates with beneficial properties are still being sought. Many are isolated from fermented plant material. Plant-associated *L. plantarum* are also a promising agent for the biological control of a wide range of plant diseases [26,27]. The condition for industrial use of strains is their correct identification and wide characterization. It should not be forgotten that the growth of *L. plantarum* can be as well undesirable. In the brewing industry, it could cause beer spoilage, which is caused by viable, VBNC (viable but nonculturable) cells growing in beer or in biofilms [28]. Therefore, its quick and accurate detection can prevent the loss of profits and consumer confidence.

Correct identification of microorganisms is an essential element of microbiological research. It is important to choose a suitable technique that will ensure obtaining reliable results. Due to the close relationship between lactic acid bacteria and the great diversity of the *L. plantarum* species, its detection and identification using traditional methods may be insufficient. Thus, this review will focus on methods for the detection and identification of plant-associated *L. plantarum* using molecular methods.

## 2. Culture-Dependent Methods

The first step in culture-dependent methods is the isolation of microorganisms on culture media. As a standard, for the isolation of lactic acid bacteria, in particular lactobacilli, the MRS medium (De Man, Rogosa, and Sharpe) or its modifications are used. For example, the addition of catalase to the MRS medium has been shown to accelerate and increase the growth of beer spoilage LABs, including *L. plantarum* [29]. However, it still shows a low degree of selectivity, so not only lactobacilli can grow on it. Attempts are being made to create selective media, but they tend to focus on *L. plantarum* isolated from specific environments such as yogurt [30] or feces [31]. Since different species of lactobacilli have similar nutritional requirements [30], so far there is no universal selective medium that allows the cultivation of only *L. plantarum*.

Due to the wide industrial use of *L. plantarum* strains, there is a need to create/optimize culture media in order to enhance its biomass [32,33,34,35,36]. In this way, the production of individual components is increased, which reduces the time and costs of the process. Low-cost industrial wastes such as cheese whey/whey permeate [37,38], corn steep liquor [38,39,40], liquid acid protein residue of soybean [39], or gelatinized starchy waste [40] have been tried for this purpose. Attempts were also made to use genome-scale metabolic models to design culture media that would better suit the nutritional requirements of *L. plantarum* [41,42]. In fact, there may be inconsistencies between predictions and actual microbial growth. The tests described above are currently being developed for specific strains. Perhaps the knowledge gained from these studies will allow in the future to create a more specific medium for the isolation of *L. plantarum*.

Depending on the purpose of the research and financial resources, after strain isolation and its depositing, various methods are used to identify them. They are presented in the following subchapters.

### 2.1. 16S rRNA Gene Sequencing

The most common molecular marker used to detect and identify bacteria, including *L. plantarum*, is the 16S rRNA gene. Woese et al. [43,44] were the first to employ it and proposed its use as a “molecular chronometer”. Over time, analysis of the 16S rRNA gene replaced even the earlier taxonomy of bacteria based on phenotypic characteristics (phenetics) and became the gold standard for identifying prokaryotes.

16S rRNA encodes a small subunit of the bacterial ribosome, hence it is a component of all bacteria. It is approximately 1500 bp long and consists of 9 variable regions (V1-V9) interspersed with conservative regions [45]. The variable regions reflect the phylogenetic distance between microorganisms, while the conserved regions, slowly changing in the process of evolution, enable the design of universal primers for all bacteria. Due to its extensive use as a molecular marker, databases are full of reference sequences, which improves the accuracy of taxonomic assignment and facilitates analysis.

The choice of primers is crucial for the obtained results. Routinely, the entire 16S rRNA gene is sequenced. Universal primers 27F (5′-AGA GTT TGA TCC TGG CTC AG-3′) and 1492R (5′-CTA CGG CTA CCT TGT TAC GA-3′) [46] are the most commonly used for this purpose. Specific primers for former *Lactobacillus* genus were also developed [47].

Years of research have shown that 16S rRNA gene as a molecular marker has some limitations. One of them is its variable copy number. In silico analyses revealed that most bacterial genomes contain several copies of the 16S rRNA gene, which may differ in sequence [48]. The more copies of a gene a microorganism has, the more diverse its sequences are [49]. In regard to *L. plantarum*, it has 5 copies of 16S rRNA gene [50,51], which in the genome of BCC9546 strain occurs in four sequence variants differing ≤0.26% [51]. It probably would not cause misidentification. The problem, however, is that the gene evolves too slowly, so it shows low resolution. For this reason, *L. plantarum* and its sixteen closely related species are indistinguishable, showing ≥99% sequence similarity [52].

Despite all above, 16S rRNA gene is still the most commonly used marker to identify bacteria, including *L. plantarum*, at least in the early stages of research. However, additional tests are necessary to identify them accurately.

### 2.2. Housekeeping Genes Sequencing

It has been shown that in the case of LAB identification, especially *L. plantarum*, analysis of housekeeping genes is more accurate. The housekeeping genes encode proteins responsible for the basic cellular metabolism and thus are constitutively expressed in every cell regardless of the conditions [53]. Like the 16S rRNA gene, they are present in every bacterium, but evolve faster, making them more variable and with a greater degree of resolution [54]. In addition, there are usually only one or two copies of these genes in the genome [55]. However, when housekeeping genes are used as molecular markers, the exact identification threshold of bacteria is unclear [54].

Choosing the right housekeeping gene as a molecular marker is crucial to obtain reliable results. Several of them are used to identify *L. plantarum* (Table 1). Recently, Pérez-Díaz et al. [56] compared the efficiency of sequencing the *recA*, *dnaK*, *pheS,* and *rpoA* genes for this purpose. The frequently used *recA* gene turned out to be not as good a marker as *pheS,* which gave more discriminatory results. Huang et al. [57] in silico, by comparing the genome sequences of different LAB species, selected another housekeeping gene—*mutL*, as a promising molecular marker for *L. plantarum* group. Based on its sequence, they developed also species-specific primers (Table 1). Their further research shows that both whole and species-specific *mutL* sequencing enables accurate differentiation and detection of *L. plantarum* and related taxa. A different approach was taken by Lee et al. [58]. They designed *L. plantarum*-specific primers targeted to transcriptional intergenic spacer region (ISR) between the *groES* and *groEL* genes, noting its similarity to an internal transcribed spacers region of 16S and 23S rRNA. The developed primers allowed for a better characterization of the species than the sequencing of the 16S rRNA gene, which was also carried out. This confirms the low resolution of the 16S rRNA gene as a marker of *L. plantarum.*

### 2.3. MultiLocus Sequence Typing (MLST)

MultiLocus Sequence Typing (MLST) is a widely used method for identification and typing of LAB [62]. It involves comparative sequence analysis of several housekeeping genes resulting in a unique allelic profile of the microorganisms. The determination of relatedness of isolates is based on comparing their allele profiles [59]. MLST is considered a precise technique, the results of which can be easily compared/exchanged between different laboratories [63].

In the case of *L. plantarum*, polymorphisms of genes: *pgm* (phosphoglucomutase), *ddl* (D-alanine-D-alanine ligase), *gyrB* (B subunit of DNA gyrase), *purK1* (ATPase subunit of the phosphoribosylaminoimidazole carboxylase), *gdh* (glutamate dehydrogenase), *mutS* (DNA mismatch repair protein) [26,62], and additionally *tkt4* (transketolase) [56] are usually tested. In addition, genes: *recA*, *pheS*, *pyrG* (CTP synthetase), *uvrC* (excinuclease ABC subunit C), *clpX* (ATP-dependent protease ATP-binding subunit ClpX), *groEL* (chaperonin GroEL)*, murC* (UDP-N-acetylmuramyl tripeptide synthase), and *murE* (UDP-Nacetylmuramoylalanyl-D-glutamate-2, 6-diaminopimelate ligase) were used for this purpose [60,63]. However, there is no common, standardized set of genes used for analysis. Appropriate selection of genes allows for obtaining acceptable identification power [59]. In addition, MLST analysis requires a lot of work, time, and costs. To overcome these disadvantages, it has been developed next-generation MLST (NGMLST) [64] or nanoMLST [65]. The use of next-generation or third-generation sequencing allowed the accuracy of the analysis to be maintained while reducing its duration and making it more cost-effective. However, there are no available studies of this type that include the analysis of lactic acid bacteria.

### 2.4. Fingerprinting Methods

Fingerprinting methods are techniques for differentially genotyping microorganisms that differently produce a variable number of DNA fragments of different sizes (fingerprint). For *L. plantarum*, the most common method used is already mentioned MLST as well as random amplified polymorphic DNA PCR (RAPD-PCR), repetitive element palindromic PCR (rep-PCR), PCR restriction fragment length polymorphism (PCR-RFLP) and pulse field gel electrophoresis (PFGE). Compared to MLST, these methods are not useful for deep phylogenetic reconstructions, therefore they are not strictly used for the identification of microorganisms. They are, however, excellent tools for distinguishing between different strains of the species. Thus, they are helpful in assessment of genetic diversity and initial characterization of strains in order to reduce the number of tested isolates [66,67]. In addition, they are used to track the presence of intentionally added strains, e.g., during the fermentation process [68]. The RAPD-PCR method was also used to determine the geographical origin of plant-related strains of *L. plantarum*, but no clear link between profiles and origin was found [69].

The RAPD-PCR does not require knowledge of the target sequences. This method is based on the use of one short primer with a random sequence, which during PCR binds to different sites on the genomic DNA. In this way, DNA fragments are generated, which are then electrophoretically separated. As a result, a strain-specific genetic fingerprint of the microorganism is obtained. Various primers are used to genotype *L. plantarum* (Table 2), which differ in their discriminatory power. For this reason, to increase the reliability of the research, several reactions are usually run in parallel using different primers.

The rep-PCR method is analogous to RAPD, except that in this case, the primer is a repeated palindromic sequence, such as: (GTG)_5_ [56,66,68] or (GACA)_4_ [70]. The research conducted by Pérez-Díaz et al. [56] showed that in case of *L. plantarum* and *Lactiplantibacillus pentosus* typing, rep-PCR-(GTG)_5_ has the most intraspecies discriminatory power compared to RAPD-PCR (LP1, OPL-05, M14, and COC primers), as well as MLST and sequencing of the *recA*, *dnaK*, *pheS,* and *rpoA* genes.

The RAPD-PCR and rep-PCR methods are relatively cheap, fast, and easy to perform, but the problem could be with repeatability and reproducibility of the analysis. These techniques are sensitive to varying DNA quantity and quality as well as PCR and electrophoresis conditions [75,76]. Hence, a highly standardized laboratory protocol is required, and additionally, these methods are not suitable for transferring and comparing the band profiles of different experiments.

The PCR-RLFP is a ribotyping method that consists of cleavage of the 16S rRNA amplicon with selected restriction enzymes. The band profile generated in this way should be species-specific. More than one restriction enzyme is used to uniquely identify microorganisms, in the case of LAB for example *Alu*I and *Mbo*I [61] or *Hae*III and *Taq*I [65]. Since PCR-RFLP is based on the analysis of polymorphic sites located on the 16S rRNA gene, it is crucial to use appropriate restriction enzymes. Laref and Belkheir [77] in silico showed that closely related species: *L. plantarum*, *L. paraplantarum*, and *L. pentosus* could be distinguished using *Muc*I, *Nsp*I, and *Tsp*DTI endonucleases. Nevertheless, the PCR-RFLP is a useful tool for only determining the interspecies diversity of LABs, hence it is rarely used [65].

PFGE, unlike other techniques discussed in the article, is a non-PCR-based typing method. It consists of immobilizing bacterial genomic/plasmid DNA in agar, cutting it with restriction enzymes (rare cutters) and separating them in agarose gel. Due to the conditions of the pulsed field of electrophoresis, the fragments are separated based on their sizes, but they are also periodically reoriented, resulting in better separation [78,79]. Adesulu-Dahunsi et al. [80] studied the genetic diversity of *L. plantarum* isolated from Nigerian fermented food, including cereal products. They digest genomic DNA with *Apa*I and *Sfi*I endonucleases. *Sfi*I was more suitable for creating unique PFGE patterns. The same enzyme was used by Lopez et al. [74] for typing *L. plantarum* isolated from musts and wine samples. Moreover, their study shows that *Sfi*I-PFGE shows a greater discriminating power for *L. plantarum* strains than RAPD-PCR using primers LP1 and OPL-05. Due to its high resolution and repeatability, PFGE is considered the gold standard for typing microorganisms [78]. However, it is labor-intensive, time-consuming, and requires high qualifications [81].

### 2.5. Whole Genome Sequencing (WGS)

The first complete genome of *L. plantarum* was sequenced by Kleerebezem et al. [82]. Since then, 825 genomes are deposited in the GeneBank NCBI database [22]. The advent of WGS and its rapid acceleration in recent years has shifted bacterial taxonomy from 16S rRNA-based to genome-based classification [83]. This is how Zheng et al. [84] reclassified the genus *Lactobacillus*. For this purpose, a polyphasic approach was used, classifying bacteria not only on the basis of genetic criteria including core genome phylogeny, average amino acid identity, and clade-specific signature genes but also considering physiological criteria and ecology of organisms.

WGS distinguishes even closely related species, but it provides more than just taxonomic information. It allowed us to explore and understand the evolution of microorganisms their interactions and adaptations [24,85]. It also increased knowledge of the molecular basis of their metabolism and stress responses. In addition, it allowed assessing their safety by detecting virulence, biocides, and antimicrobial substance resistance genes. Although *L. plantarum* is generally considered safe, infection is still possible, especially in immunocompromised individuals [86]. Thus, the analysis of safety is required before using every strain as a pure culture or probiotic.

Because WGS is time-consuming and still expensive it is not yet a routinely used method. In this way, mainly valuable strains with high biotechnological potential are identified and characterized. Constantly developing technology lowers the cost of sequencing, so in the near future, WGS may become the new gold standard for classifying and identifying microorganisms [87]. However, WGS generates huge amounts of data, the analysis of which takes a long time and requires space for their storage [88]. This may involve additional costs. Additionally, making such large data available to online bioinformatics databases can be a challenge. This requires high-capacity computers and/or fast, reliable Internet connections, which can be a problem in developing countries [89]. Nonetheless, thanks to the comparative analysis of different genomes deposited in databases, it is possible to get to know microorganisms in more depth, and thus more precisely design new molecular tools that will effectively serve, for example, their identification and detection.

## 3. Culture-Independent Methods

It is estimated that using culture-dependent methods, only about 1% of bacteria are isolated from a given environment [90]. This is supported by the fact that most of the bacterial phyla have been uncultured [91]. The main limiting factor is the culture media used. In addition, microorganisms in the environment can be in a number of states, and only some of them enable active replication. An example is the VBNC (viable but nonculturable) state in which bacteria, despite being viable and active, are unable to grow on the media. This is a known survival strategy of non-sporulating bacteria. In addition, *L. plantarum* is able to enter into the VBNC state [28]. Therefore, for industries where they can harm the process, such as beer production, direct detection and identification of LABs by culture-independent methods can be particularly useful.

### 3.1. Real-Time PCR (qPCR)

Real-time PCR (quantitative PCR, qPCR) is a rapid, sensitive, and specific method used for the detection, quantification, and typing/identification of microorganisms [92]. The amplification of the product is monitored in real time, thanks to the use of fluorescent DNA dyes and/or probes which are added to the PCR mixture before amplification [93] The method enables the quantification of the product in two ways: absolute or relative. Absolute quantification requires a calibration curve against which the results are compared (with the exception of digital PCR). It is applicable to microbial load or copy number quantification. In the case of relative quantification, another foreign reference sample is used to assess the amount of product. It is performed for gene expression analysis [94].

qPCR can be used as both a culture-dependent method, by analyzing DNA isolated from pure cultures [95,96,97,98], and a culture-independent method, by analyzing a pool of genomic DNA isolated from a sample [99,100,101]. In the second case, however, there is no distinction between living and dead cells [102]. In both cases, detection and identification of *L. plantarum* could be completed using species-specific primers targeting to 16S rRNA gene [103] or already discussed housekeeping genes [101].

Recently, several species-specific primers have been designed for qPCR applications (Table 3). Xiong et al. [99] designed a set of primers targeted to representative sequences in *L. plantarum* NCU116 (*tal* gene). The pair Ftal1/Rtal1 turned out to be sufficient for the detection and identification of *L. plantarum*. In addition, the comparison of the number of microorganisms obtained by qPCR and plate cultures gave comparable results, so these primers also allow for reliable cell quantification even directly in the food samples. The other primers were designed based on a comparative analysis of the *L. plantarum* genomes. On this basis, Kim et al. [95] designed primers for several former *Lactobacillus* species. In the case of *L. plantarum*, the marker was the gene encoding the cell wall anchor domain protein with the LPXTG motif. Additionally, Jin et al. [96] designed *L. plantarum* subsp. *plantarum*-specific primers targeting the LPXTG gene. For *L. plantarum* subsp. *argentoratensis bspA* gene was used. In another study, also Kim et al. [97] designed *L. plantarum* subspecies-specific primers based on the *ydiC* gene sequence. In all cases, all primers used allowed for the correct identification of species or subspecies level, and the specificity of the primers was confirmed using reference strains. Using primers developed by Kim et al. [97], Choi et al. [98] analyzed in silico their usefulness in identifying *L. plantarum* subsp. *plantarum*. Next, they compared the amplification efficiency of the *ydiC* gene using digital droplet PCR (ddPCR) and qPCR. ddPCR represents the third generation of qPCR. Briefly, it allows to performance of qPCR in a droplet containing a single template. Both methods were suitable for *L. plantarum* subsp. *plantarum* identification, but ddPCR was more sensitive than qPCR (has a 10 times lower limit of detection). Thus, it could be used to detect *L. plantarum* in foods where they are found in small amounts, e.g., as contaminants in beer.

A variant of qPCR, multiplex PCR, has been successfully used to detect lactic acid bacteria causing beer spoilage, including *L. plantarum*. It allows the amplification of multiple templates in a single PCR reaction. In this case, molecular markers could be genes responsible for hop resistance, e.g., horA, horC. Their presence is associated with the ability of bacteria to spoil beer. It is hypothesized that these genes may be transferred by horizontal gene transfer [104]. Haakensen et al. [105] by horA-specific qPCR studied the occurrence of this gene in various lactic acid bacteria. In all *L. plantarum* strains of brewing origin and strains isolated from corn silage tests confirmed the presence of the horA gene. This may confirm the potential of *L. plantarum* to contaminate beer.

The discussed examples show that the qPCR method is fast and effective in the study of *L. plantarum*. Their analysis is facilitated by commercial qPCR kits appearing on the market, also targeting this microorganism. However, PCR inhibitors that may be bound to environmental samples may present problems in the analysis, e.g., causing skewed quantification. DNA purification or separation can help, but this leads to the loss of some material. The solution is also the use of inhibitor-tolerant DNA polymerase [106]. Moreover, qPCR equipment is expensive compared to classic thermal cyclers. For this reason, less affluent laboratories can adapt the designed specific primers to classical PCR and simply visualize the target product on the gel. However, this will involve more work.

### 3.2. 16S rRNAs and Shotgun Next and Third Generation Sequencing

The development of sequencing techniques resulted in the emergence of next-generation sequencing (NGS) and later third-generation sequencing (TGS) methods on the market. Both technologies enable high-throughput and parallel sequencing of multiple samples simultaneously. The main difference is the length of the analyzed fragments of nucleic acids—NGS allows short reads sequencing (on average 150–300 bp) while TGS long reads sequencing (up to 3 Mb) [107]. Several NGS and TGS platforms are available on the market. Each of them is based on different technologies, as a result of which the systems differ in, among others, sequencing efficiency or cost. Both NGS and TGS are widely used in microbiological research. With the exception of the whole genome sequencing discussed above, these technologies are mainly used to study the microbial community in a given environment through 16S rRNA amplicon analysis or metagenomic sequencing.

The analysis of the microbiome based on the 16S rRNA gene enables the study of its structure in terms of presence and relative abundance [108]. In this way, the bacterial community was examined, among others: *Eichhornia crassipes* (NGS) [109], Peruvian maize-based fermented beverage *chicha* (NGS) [110], kimchi [111], sourdoughs (NGS) [112], whole crop corn silage fermentation (TGS) [113]. However, it is not a good method in the context of the detection and identification of *L. plantarum*. As already mentioned, the 16S rRNA gene is not a good taxonomic marker for it, especially in the case of NGS, which sequences only a short fragment of the gene. This method may also fail to detect some microorganisms. According to the research by Durazzi et al. [114], 16S rRNA sequencing detects only part of the microbiota community revealed by shotgun sequencing (both studies were performed on the same NGS sequencer). In addition, several phyla were less abundant in the analysis of 16S rRNA in comparison to shotgun sequencing. In the case of 16S rRNA analysis, the primers used may affect the results obtained.

The shotgun method enables direct sequencing of the genomes of all microorganisms present in the tested sample. To make this possible, genomes are randomly cut into smaller fragments, which are then sequenced. Depending on the technology used, the fragments are shorter or longer. The sequenced fragments are then assembled by computers, which ordered them by finding the overlapping ends of the sequences [115]. Unlike sequencing of the 16S rRNA amplicons, shotgun sequencing provides insight into both the taxonomy and metabolism of complex microbial communities [116]. It has been used in metagenome research of various plant environments such as: *Arabidopsis thaliana* leaf (NGS) [117], apple fruit (NGS) [118], alcoholic beverages made of agave sap (NGS) [119], Kombucha tea (NGS) [120], naturally fermented soybean food (TGS) [121], traditional Chinese plant fermented food *zha-chili* (TGS) [122]. Research by Meslier et al. [116] showed that shotgun sequencing performed with TGS has an advantage in the analysis of complex microbial communities over the same analysis performed with the NGS method. However, they require careful preparation of the library.

Shotgun sequencing provides more reliable information on the community of *L. plantarum* in the environment than 16S rRNA sequencing. However, since both methods are based on DNA analysis, it is not possible to distinguish between living and dead microorganisms. Even if a bacterium with interesting properties is detected in the environment during the metagenomic analysis, it cannot be used because it is not cultured. For this reason, these methods are often combined with culture-dependent analysis, resulting in a broader understanding of microbial communities.

## 4. Conclusions

*Lactiplantibacillus plantarum* is a bacterium that fascinates scientists but also often poses challenges to them, including its detection and identification. A widely used genetic marker, 16S rRNA, has too low of a discriminating power for the *L. plantarum* group, which may lead to its incorrect classification. Housekeeping genes appear to be a more appropriate marker, but there is no universal protocol for their use. Among the culture-dependent methods discussed, the most reliable for identifying *L. plantarum* seemed to be still expensive and not always achievable whole genome sequencing. The increasing use of this technique in combination with in silico analysis may in the future allow for the development of cheaper and more accurate tests for *L. plantarum*, possibly also selective culture media. With regard to the culture-independent methods, quantitative PCR is the most suitable for the detection of *L. plantarum* direct in the sample. 16S rRNA and shotgun sequencing using next- and third-generation sequencers are better for studying microbial communities than single-species detection and identification.

## Figures and Tables

**Table 1:** Housekeeping genes used for *L. plantarum* identification.

Target Gene	Function	Primer Name	Primer Sequence (5′ → 3′)	References
*rpoA*	α-subunit of RNA polymerase	rpoA-21-F	ATG ATY GAR TTT GAA AAA CC	[56]
		rpoA-22-R	ACY TTV ATC ATN TCW GVY TC	
*pheS*	phenylalanyl-tRNA synthase	pheS-21-F	CAY CC NGC HCG YGA YAT GC	[56]
		pheS-22-R	CCW ARV CCR AAR GCA AAR CC	
*dnaK*	heat shockresponse	Lpdnak-500F3 ^1^	CCG TTC TTR TCR ATR TCR AA	[56]
		Lpdnak-1710R5 ^1^	GAA AYY CAA GTY GGH GAA GT	
*recA*	DNA repair and maintenance	planF ^2^	CCG TTT ATG CGG AAC ACC TA	[26,56,59,60,61]
		pREV ^2^	TCG GGA TTA CCA AAC ATC AC	
*mutL*	DNA mismatchrepair	LpmutL-F	TSG AYG TSA AYG TKC AYC C	[57]
		LpmutL-R	ATG YGG RCA RTT RAA NGG AT	
		spLplan-F ^2^	GCG RTT GTT CCG TCA GAA T	
		spLplan-R ^2^	CTT GCA GCC GTG CTG GTT T	
ISR of *groESL*	intergenic spacer region	Lplan-F ^2^	GGA CAA AAG TTG ACC CCA GCG	[58]
		Lplan-R ^2^	ACC GTT GCA GTA GTC GTC CC	

^1^*L. plantarum* group specific; ^2^ *L. plantarum* specific.

**Table 2:** Primers used for *L. plantarum* RAPD fingerprinting.

Primer Name	Primer Sequence (5′ → 3′)	References
M13	GAG GGT GGC GGT TCT	[67,69,70,71,72,73]
M14	GAG GGT GGG GCC GTT	[56]
LP1	ACG CGC CCT	[56,74]
OPL-05	ACG CAG GCA C	[56,67,74]
COC	AGC AGC GTG G	[56]
P1	GCG GCG TCG CTA ATA CAT GC	[73]
P2	ATG TAA CGC C	[67]
P4	CCG CAG CGT T	[67,73]
B10	CTG CTG GGA C	[73]

**Table 3:** *L. plantarum* species and subspecies-specific qPCR primers.

Target Gene	Function	Primer Name	Primer Sequence(5′ → 3′)	Product Size [bp]	References
*LPXTG*	LPxTG-motif cell wall anchor domain protein	Plantarum-F	GCT GGC AAT GCC ATC GTG CT	147	[95]
		Plantarum-R	TCT CAA CGG TTG CTG TAT CG		
		T1PL186F ^1^	ACC CCC GTT CCG TCA GA	186	[96]
		T1PL186R ^1^	ATC ACC GCT TCC CCG CTC ATT		
*bspA*	leucine-rich repeat surface protein	LPA187F ^2^	GCA TCC CGA CGC TAC TAC ACA	187	
		LPA187R ^2^	GAT TTT ATT TGC GTC CCA CTC C		
*ydiC*	hypothetical protein	Plantarum_F ^1^	CGG CAA CAA GCC ACT AAA CT	120	[97]
		Plantarum_R ^1^	TTC TTG ATG GCC CGG GTG TT		
		Argentoratensis_F ^2^	TTC TTG ATG GCC CGG GTG TT	143	[97,98]
		Argentoratensis_R ^2^	GGC TGG ACC ATG GCT AAG AA		
not stated	hypothetical protein	CMC1F	AGT TTG CCA CAT ATT AGG AAG AGA	112	[100]
		CMC1R	AGG CTC TAA GGG CTA CCT ATA C		
*tal*	unknown	Ftal1	AAC ATT TCG CGG AAC TTG GTT	160	[99]
		Rtal1	ATC ATC TCT TCG GCC TTG GT		

^1^*L. plantarum* subsp. *plantarum* specific, ^2^ *L. plantarum* subsp. argentoratensis specific.

## Data Availability

Not applicable.
